# Determinants of willingness to undergo breast cancer prophylactic examinations in Polish women

**DOI:** 10.3389/fpubh.2025.1583414

**Published:** 2025-09-30

**Authors:** Amelia Ciuba, Marta Kulpa, Aneta Nitsch-Osuch

**Affiliations:** ^1^Department of Social Medicine and Public Health, Doctoral School, Medical University of Warsaw, Warsaw, Poland; ^2^Department of Health Psychology, Medical University of Warsaw, Warsaw, Poland; ^3^Department of Social Medicine and Public Health, Medical University of Warsaw, Warsaw, Poland

**Keywords:** breast cancer prevention, individual determinants, internal-external control, health behavior, public health

## Abstract

**Background:**

Breast cancer is a global health issue affecting all countries regardless of economic development status. In Poland, the risk remains high: over the past 20 years, breast cancer incidence has increased by 60%, and mortality has risen by 30%. Although prevention options are limited, lifestyle modifications and regular medical check-ups can help reduce risk. This study aimed to identify key factors influencing Polish women’s willingness to undergo breast cancer screening.

**Material and methods:**

The study surveyed 407 women aged 45 to 69 between 2021 and 2022 and reported using the STrengthening the Reporting of OBservational studies in Epidemiology (STROBE) checklist. The research tool included an author-delivered questionnaire, the Multidimensional Health Locus of Control (MHLC) scale, the Health Behavior Inventory (HBI), and the Mini-Coping Orientation to Problems Experienced (Mini-COPE) inventory.

**Results:**

Women were more likely to participate in screening if they were older, married, had children (although having an additional child was associated with a lower likelihood of screening), used oral contraception, had received education on breast cancer prevention, had higher average intensity in the Health Behavior Inventory and scored significantly higher on the author’s scale, and demonstrated an external health locus of control. Additionally, women who reported attending prophylactic examinations were significantly more likely to express a need for emotional support.

**Conclusion:**

Further research is necessary to gain a deeper understanding of the factors that motivate women to engage in health-promoting behaviors and to develop targeted interventions. The findings suggest that women who are well-informed about breast cancer prevention and feel comfortable accessing preventive services are more likely to undergo regular screening.

## 1 Introduction

Breast cancer is the most frequently diagnosed cancer and the leading cause of cancer-related mortality among women worldwide. In 2022, there were 2,296,840 new cases (age-standardised rate (ASR) 46.8/100,000) and 666,103 deaths (ASR 12.7/100,000) (1). This disease constitutes a significant global public health burden, affecting countries irrespective of their economic development status. In Poland, 21,079 new cases (ASR 59.82/100,000) were reported in 2021, representing 24.2% of all malignant neoplasms diagnosed in women. During the same period, 6,406 deaths were recorded (ASR 13.6/100,000), accounting for 14.9% of all female cancer-related fatalities (2). Over the past two decades, the incidence of breast cancer among Polish women has increased by 60%, while mortality has risen by 30%. These trends underscore the paramount importance of effective prevention and early detection strategies to mitigate the disease’s impact.

Statistically, one in eight women will develop breast cancer during her lifetime. When detected at an early stage, a woman’s chance of achieving remission is nearly 80%. Despite this, mammography screening rates in Poland remain low; in 2024, only 31% of eligible Polish women underwent screening, far below the recommended 70–80% threshold necessary for population-level effectiveness. Mammography remains the most effective method for early detection, thereby increasing the likelihood of remission and reducing the need for intensive treatment (3).

Cancer prevention encompasses activities aimed at reducing the risk of disease onset by enhancing overall health (primary prevention) and detecting disease at an early, preclinical stage (secondary prevention). In the case of breast cancer, primary prevention options are limited but important. Numerous studies have identified several factors associated with an increased risk of breast cancer, including a diet rich in saturated fat, alcohol consumption, cigarette smoking, excessive body weight, a sedentary lifestyle, physical inactivity, prolonged use of hormone replacement therapy, having a first pregnancy after the age of 30 or never having a full-term pregnancy, and not breastfeeding (4–6). It is possible to reduce the risk of death from breast cancer by adopting a healthy lifestyle and attending regular medical check-ups. For early detection, undergoing diagnostic tests is crucial, with regular mammography being the most important screening method for breast cancer. In Poland, as part of breast cancer prevention efforts, the National Health Fund invites all insured women aged 50–69 to undergo free mammography screening every two years. Since November 1, 2023, this program has been extended to include women aged 45–74 (7). For younger women, an annual breast ultrasound is recommended due to mammography’s reduced sensitivity in dense breast tissue. Regardless of age, regular breast self-examination and prompt medical consultation for any concerns are advised. Physicians may recommend mammography outside the screening program for individuals at high risk, who are often covered by insurance.

The effectiveness of promoting pro-health attitudes depends on healthcare quality, screening accessibility, costs, and individual factors such as gender, education, residence, and family status. Psychological and social determinants, including health perception, locus of control, and self-efficacy, also influence health behaviors. Addressing these multifaceted factors enhances the success of health education initiatives targeting individuals and communities.

Barriers to screening participation include psychological factors such as shame, fear of diagnosis or pain, and discomfort during procedures, as well as practical issues such as time constraints, perceived good health, and a lack of familial or social habits that support screening. Systemic obstacles include healthcare accessibility, quality of doctor-patient relationships, appointment wait times, and out-of-pocket costs. Understanding these barriers is essential for designing effective interventions to improve screening uptake.

This study examines the combined influence of cognitive and emotional–behavioral factors on women’s participation in breast cancer screening. Cognitive components, such as health beliefs and locus of control, were assessed using the Multidimensional Health Locus of Control version B (MHLC-B) questionnaire, while emotional–behavioral factors, including stress coping and health behaviors, were evaluated through the Health Behavior Inventory (HBI), Mini-Coping Orientation to Problems Experienced (Mini-COPE), and an author-designed questionnaire. This comprehensive approach enables the identification of risk groups and the psychological mechanisms underlying low participation rates, highlighting the need to tailor educational and prevention programs to individual beliefs and coping strategies.

Health behaviors encompass actions affecting physical, mental, and social well-being and include habits, attitudes, and values recognized within social contexts (8). According to Marc Lalonde, health behavior is the most significant factor influencing human health. Lalonde’s model identified that health is determined by multiple factors: healthcare organization (10%), biological and genetic factors (16%), environmental factors (21%), and, most importantly, health behaviors and lifestyle (53%). Key behaviors include physical activity, diet, sleep hygiene, substance use, sexual health, and participation in preventive measures (9). Early modeling of health behaviors, particularly by parents, and cultural, social, religious, demographic, and socioeconomic factors further shape individual health practices (10, 11).

Motivations for undergoing prophylactic examinations (beyond those required by employers) vary depending on general attitudes toward health. Individuals with an internal locus of control tend to take greater responsibility for their health, are aware of the impact of their decisions, and are more likely to engage in health-promoting activities. Additionally, those with a family history of increased risk for diseases such as cancer are more likely to participate in preventive examinations, driven by a desire to monitor their health and reduce the risk of developing severe illness at a late stage. In contrast, individuals with an external locus of control believe they have little influence over their health and expect others to take care of them. They often attribute health problems to external factors. For these individuals, health-seeking behavior is primarily motivated by a sense of threat or fear of illness, usually triggered by alarming symptoms, such as a lump in the breast, persistent malaise, or pain. As a result, they tend to seek prophylactic examinations much later than those with an internal locus of control. Although numerous preventive initiatives, including social campaigns and other health educational initiatives in Poland, have raised awareness about the importance of breast self-examination and mammography, this awareness does not always translate into regular participation in such examinations. The frequency of preventive examinations is influenced by a variety of individual and broader factors (12). Beyond nationwide campaigns, breast cancer prevention is also promoted through more localized efforts, often taking the form of community, regional, or private initiatives. For example, on World Cancer Day, oncology centers offer health talks and free screenings, such as mammograms for insured women aged 45 to 74. Additionally, oncology centers and non-governmental organizations (NGOs) conduct health education workshops and training sessions that emphasize breast self-examination, raise awareness about the realities of cancer, and highlight the significance of routine screenings and early intervention. Furthermore, employers organize educational programs during Pink October, and schools offer similar programs aimed at children and adolescents.

In Poland, the population-based breast cancer screening program, launched in 2006, has struggled with low coverage, declining from 42.23% in 2016 to 33.64% in 2021, with only slight improvements following the coronavirus disease 2019 (COVID-19) pandemic restrictions (35.33% in 2022, 36.14% in 2023, and 32.46% in 2024). These data highlight the importance of identifying factors influencing women’s awareness and motivation to participate in screening. Understanding these determinants is crucial for developing targeted health promotion strategies to increase mammography uptake and ultimately reduce breast cancer mortality.

The National Oncology Strategy (NOS), adopted in 2020 as a multi-year program for 2020–2030, aims to reform the Polish oncology system by reversing adverse epidemiological trends, improving therapy effectiveness, and aligning care with patient needs. Reports on the implementation of the NOS highlight several significant challenges and recommendations. First, there is an emphasized need to strengthen educational and communication efforts to increase women’s engagement in regular preventive screenings. Second, attention is drawn to the necessity of identifying factors that influence women’s awareness and motivation, including the need to modify strategies for reaching health promotion audiences. The reports also recommend the development of health education incorporating psychological components. Third, it has been confirmed that the accessibility of preventive examinations, along with ensuring patients’ sense of safety and comfort, is crucial for increasing the regularity of participation in screening programs. This study addresses the challenges of educational and promotional fragmentation, thereby supporting more effective cancer prevention in Poland.

A review of the literature indicates that there are limited studies on the Polish population that simultaneously analyze knowledge, attitudes, and psychological and behavioral components affecting women’s participation in breast cancer prophylactic examinations using standardized questionnaires, such as MHLC, HBI, and Mini-COPE. This gap is further underscored by international literature, where existing research often relies on proprietary questionnaires or singular standardized tools, resulting in fragmented insights. There is a clear and critical need for multidimensional research frameworks that holistically integrate cognitive, affective, and behavioral factors across diverse populations. Addressing this gap, the present study employs a combination of standardized tools to provide a comprehensive perspective on the determinants of women’s participation in breast cancer screening within the Polish context. It is important to note that the findings presented here are derived from exploratory research, designed to generate initial insights and hypotheses that will guide future studies and health interventions.

The study aimed to identify the key determinants influencing the willingness of Polish women to participate in breast cancer prophylactic examinations.

## 2 Materials and methods

### 2.1 The study group

The study included 407 women aged between 45 and 69 (mean 54.86 ± 6.718). It was conducted as part of a doctoral thesis at the Medical University of Warsaw, Poland, between March 2021 and May 2022. Paper-based questionnaires were distributed by staff at adult education centers, whereas the online survey was disseminated among employees of a large state-owned corporation. The study was conducted within the city of Warsaw, including participants who utilize modern technologies (the Internet). Therefore, it was assumed that the estimated proportion of women attending screening in this sample would be higher than that of the general Polish population. Participation in the survey was voluntary and anonymous. Due to the nature and scope of the study, a convenience sampling method was used. The study was reported using the STrengthening the Reporting of OBservational studies in Epidemiology (STROBE) checklist (Appendix 1; Table 1).

**Table 1:** Comparison of respondents who confirmed participation in prophylactic examinations and those who did not confirm it.

Variable	Participation in prophylactic examinations		MD (95% CI)	*p*
	Yes (*n* = 304)	No (*n* = 103)		
Age, years, me (IQR)	55.00 (50.00; 60.00)	51.00 (47.50; 60.50)	4.00 (−1.00; 6.00)	0.072^3^
City size				
Village	53 (17.4)	11 (10.9)	-	0.441
City with up to 50,000 inhabitants	56 (18.4)	20 (19.8)		
City with 50,000–150,000 inhabitants	44 (14.5)	12 (11.9)		
City with 150,000–500,000 inhabitants	23 (7.6)	7 (6.9)		
City with above 500,000 inhabitants	128 (42.1)	51 (50.5)		
Education				
Vocational	18 (5.9)	7 (6.8)	-	0.191
Secondary	74 (24.3)	34 (33.0)		
Higher	212 (69.7)	62 (60.2)		
Marital status				
Single/unmarried	17 (5.6)	13 (12.6)	-	**0.026**
Informal relationship	17 (5.6)	5 (4.9)		
Married	194 (63.8)	51 (49.5)		
Divorced	53 (17.4)	20 (19.4)		
Widow	23 (7.6)	14 (13.6)		
Employment status				
Active	253 (83.2)	81 (78.6)	-	0.285
Unemployed	3 (1.0)	1 (1.0)		
Retired	36 (11.8)	19 (18.4)		
Pensioner	12 (3.9)	2 (1.9)		
Number of children, me (IQR)	2.00 (1.00; 2.00)	1.00 (1.00; 2.00)	1.00 (0.00; 1.00)	**0.042** ^**3**^
Hormonal contraception	145 (47.7)	30 (29.1)	-	**0.001**
Healthy lifestyle	222 (74.2)	67 (65.7)	-	0.124
Knowledge on breast cancer (self-assessment)				
Very high	35 (11.5)	11 (10.8)	-	0.098
High	161 (53.0)	41 (40.2)		
Medium	95 (31.2)	43 (42.2)		
Low	9 (3.0)	3 (2.9)		
Very low	4 (1.3)	4 (3.9)		
If consulted herself, if a close relative has had breast cancer*	69 (57.5)	17 (41.5)	-	0.111
If went through the educational process on breast cancer				
Yes	138 (45.4)	32 (31.1)	-	**0.039**
No	157 (51.6)	67 (65.0)		
I do not know	9 (3.0)	4 (3.9)		
If went through education on early detection of breast cancer				
Yes	151 (49.7)	38 (36.9)	-	0.080
No	113 (37.2)	48 (46.6)		
I do not know	40 (13.2)	17 (16.5)		
Medical history of breast cancer				
Yes, I am currently in the treatment process	49 (16.1)	11 (10.7)	-	0.506
Yes, currently with treatment completed	12 (3.9)	4 (3.9)		
Yes, currently in relapse of the disease	2 (0.7)	0 (0.0)		
No	241 (79.3)	88 (85.4)		
Health Behavior Inventory				
Healthy habits nutrition, HHN [1–30], M ± SD	22.37 ± 4.22	21.48 ± 4.90	0.89 (−0.25; 2.03)	0.124^2^
Preventive behavior, PB [1–30], M ± SD	22.86 ± 4.04	20.73 ± 4.97	2.13 (0.98; 3.28)	**< 0.001** ^**2**^
Positive adjustments, PA [1–30], M ± SD	22.03 ± 3.96	21.00 ± 4.65	1.03 (0.03; 2.02)	**0.043** ^**1**^
Health practices, HP [1–30], M ± SD	20.93 ± 3.69	19.70 ± 4.62	1.23 (0.16; 2.29)	**0.024** ^**2**^
Mini-COPE				
Active coping [0–3], Me (IQR)	2.50 (2.00; 3.00)	2.50 (2.00; 3.00)	0.00 (0.00; 0.00)	0.125^3^
Planning [0–3], Me (IQR)	2.50 (2.00; 3.00)	2.00 (2.00; 3.00)	0.50 (−0.50; 0.50)	0.097^3^
Positive reappraisal [0–3], M ± SD	1.84 ± 0.74	1.93 ± 0.69	−0.09 (−0.26; 0.08)	0.298^1^
Acceptance [0–3], M ± SD	1.94 ± 0.74	1.94 ± 0.63	0.00 (−0.17; 0.17)	0.997^1^
Sense of humor [0–3], M ± SD	0.75 ± 0.63	0.86 ± 0.58	−0.11 (−0.25; 0.04)	0.150^1^
Turning to religion [0–3], Me (IQR)	1.00 (0.00; 2.00)	1.00 (0.00; 2.00)	0.00 (0.00; 0.50)	0.356^3^
Seeking emotional support [0–3], M ± SD	2.14 ± 0.80	1.94 ± 0.81	0.20 (0.01; 0.39)	**0.035** ^**1**^
Seeking instrumental support [0–3], M ± SD	2.02 ± 0.77	1.97 ± 0.79	0.06 (−0.13; 0.24)	0.544^1^
Dealing with something else [0–3], M ± SD	1.82 ± 0.78	1.86 ± 0.79	−0.04 (−0.22; 0.14)	0.654^1^
Denial [0–3], M ± SD	0.80 ± 0.76	0.98 ± 0.79	−0.18 (−0.36; 0.00)	0.053^1^
Venting of emotions [0–3], M ± SD	1.41 ± 0.69	1.48 ± 0.68	−0.07 (−0.23; 0.10)	0.425^1^
Use of psychoactive substances [0–3], Me (IQR)	0.00 (0.00; 0.50)	0.00 (0.00; 1.00)	0.00 (0.00; 0.00)	0.321^3^
Suppression of activities [0–3], M ± SD	0.71 ± 0.68	0.81 ± 0.67	−0.11 (−0.27; 0.05)	0.180^1^
Self-blame [0–3], M ± SD	1.15 ± 0.76	1.26 ± 0.76	−0.11 (−0.28; 0.07)	0.236^1^
MHLC-B				
Internal [6–36], M ± SD	24.91 ± 5.19	25.13 ± 5.92	−0.23 (−1.51; 1.06)	0.728^1^
Powerful others [6–36], M ± SD	20.41 ± 5.92	18.10 ± 5.80	2.31 (0.91; 3.71)	**0.001** ^**1**^
Chance [6–36], M ± SD	17.60 ± 6.39	18.52 ± 6.11	−0.92 (−2.42; 0.59)	0.232^1^
Pro-Health Behavior scale, Me (IQR)	4.50 (3.50; 5.50)	2.50 (1.00; 4.00)	2.00 (1.50; 2.50)	**< 0.001** ^**3**^
Awareness of prevention possibilities scale, M ± SD	11.38 ± 3.97	10.13 ± 3.72	1.26 (0.38; 2.13)	**0.005** ^**1**^

M – mean, SD – standard deviation, Me – median, IQR – interquartile range, MD – mean or median difference (yes vs. no), CI – confidence interval. Comparisons conducted with Student’s t-test^1^, Welch’s t-test^2^, Mann–Whitney U test^3^, Pearson’s chi-squared test or Fisher’s exact test^4^, as appropriate. * Proportion calculated in relation to the total number of available answers (*n* = 120 in “yes” group and *n* = 41 in “no” group). Statistical significance at *p*<0.05 is indicated in bold font.

### 2.2 Sample size calculation

The calculation of the minimum sample size indicated that 385 female respondents would be sufficient to achieve statistical significance. The size of the study group was calculated using the following assumptions: an estimated proportion of 50% women attending screening, a significance level (*α*) of 5%, a population size of 6,427,420 (women aged between 45–69 years, based on the Central Statistical Office of Poland, 2024), and a permissible margin of error of 5%.

Considering the available methods for reaching respondents and the specific location of the study, we anticipated that the study population would be more educated and more receptive to new technologies compared to the general Polish population. Based on this assumption, we estimated that approximately 50% of women within the targeted age group would participate in screening programs. This estimate was then used as the expected proportion in calculating the sample size.

Calculations were made using an online sampling calculator available at: https://www.calculator.net/sample-size-calculator.html?type=1&cl=95&ci=5&pp=50&ps=6%2C427%2C420&x=Calculate.

### 2.3 Data collection

The survey was conducted both on paper, using the snowball sampling technique, and electronically, using Computer Assisted Web Interviewing (CAWI). The original plan was to use the paper-and-pencil method and reach respondents directly. However, restrictions related to the COVID-19 pandemic necessitated that the researchers had to expand the study to include an electronic form.

### 2.4 Potential bias associated with the use of two data collection techniques (paper-based surveys and CAWI)

The number of paper-based and online surveys was approximately equal, with around 200 respondents in each group. The inclusion of the online survey was necessitated by circumstances related to the COVID-19 pandemic. The primary relationship under investigation (participation in screening) was present in both the paper-based and online (CAWI) survey groups. To assess whether the mode of survey form influenced the strength of this relationship, we treated the survey delivery method as a moderator and conducted an analysis using the PROCESS macro developed by Andrew Hayes. The results indicated that the mode of survey administration did not moderate the relationship between the variables. Consequently, it was determined that combining both groups was appropriate for subsequent analyses.

### 2.5 PROCESS macro

To determine whether the mode of survey administration (paper-based versus online) influenced the relationship between the key variables, we conducted a moderation analysis using the PROCESS macro developed by Andrew Hayes. It is a widely recognized tool that simplifies the examination of interaction effects in regression models, allowing for efficient testing of whether the strength or direction of relationships varies across different groups or conditions (13).

In the present analysis, the survey format was included as a moderator to assess if it altered the association between participation in screening and the variables of interest. The results showed no significant moderation effect, indicating that the relationship was consistent regardless of whether respondents completed the paper or the online survey. Based on these findings, data from both survey modes were combined for further analyses.

### 2.6 Bioethics committee

The study was acknowledged by the Bioethics Committee at the Medical University of Warsaw, decision number AKBE/47/2021. However, no formal protocol was registered or approved by the ethics committee before the commencement of the study. The absence of a preregistered protocol is hereby acknowledged for the sake of transparency and research integrity. No separate protocol document exists for this study.

### 2.7 Research tool

Research tools included an author-designed questionnaire and standardized questionnaires of the Psychological Test Laboratory of the Polish Psychological Association: (1) the Multidimensional Health Locus of Control Scale version B (MHLC-B) by Kenneth A. Wallston, Barbara S. Wallston, and Robert DeVellis, adapted by Zygfryd Juczynski, (2) the Health Behavior Inventory (pol. Inwentarz Zachowań Zdrowotnych, IZZ) by Zygfryd Juczynski, and (3) the Coping Orientation to Problems Experienced questionnaire (Mini-COPE) by Charles S. Carver, adapted by Zygfryd Juczynski and Nina Ogińska-Bulik.

The standardized questionnaires were purchased from the Psychological Test Laboratory of the Polish Psychological Association and were used as intended. Written permission was obtained to use the MHLC-B and Mini-COPE questionnaires in the electronic version of the survey. A paid license for the electronic use of the HBI questionnaire was obtained, as confirmed by an agreement dated 3 January 2021. The electronic research tool was password-protected, as requested by the Laboratory. The results from each questionnaire were analyzed separately based on the diagnostic key available in the test manual.

The author-designed questionnaire was created to identify demographic and sociological factors influencing women’s enrollment in preventive mammography screening in Poland. The survey consisted of 40 questions and was divided into 12 thematic areas, including: sociodemographic data (such as age, place of residence, educational level, marital status, occupational situation, mode of work, gynecological and reproductive history, lifestyle and subjective assessment of health, knowledge of breast cancer, family history and reactions to illness of loved ones, participation in screening, breast self-examination practices, opinions on the recommended frequency of examinations, opinions on barriers to preventive measures, health behaviors, and experience with breast cancer).

These twelve areas enabled the calculation of two main variables – “Pro-Health Behavior” and “Awareness of Prevention Possibilities.” The author-designed questionnaire is provided in Appendix 2, accompanied by the calculation key in Appendix 3 (Table 1), ensuring complete transparency and reproducibility. The two main scales used in this study were developed based on an extensive review of existing literature regarding breast cancer risk factors, preventive behaviors, and health education guidelines. Items incorporated into the questionnaire were selected to capture both participants’ actual health-related behaviors and their knowledge or perceptions related to breast cancer prevention.

Specifically, the Pro-Health Behavior scale comprises items that reflect concrete preventive actions and lifestyle behaviors, such as participation in mammography screenings, frequency of breast self-examinations, and levels of physical activity. These items were assigned point values corresponding to their documented protective effects or importance in reducing breast cancer risk, drawn from epidemiological evidence and established health recommendations.

Conversely, the Awareness of Prevention Possibilities scale primarily includes items that measure knowledge and beliefs regarding risk factors, early symptoms, and preventive measures. Some items were included in both scales due to their dual role, which encompasses both behavior and awareness. For instance, questions related to self-assessment of behaviors such as breast self-examination or physical activity appear in both scales. Still, they are scored differently to distinguish actual engagement from knowledge or understanding. This methodological approach reflects the conceptual distinction between *what individuals do* and *what they know or believe*, enabling a more nuanced analysis of the factors influencing preventive health behaviors.

Specifically, physical activity is represented in both scales but weighted differently: within the Pro-Health Behavior scale, points quantify the extent and regularity of exercise as a protective health behavior, whereas in the Awareness of Prevention Possibilities scale, points reflect the participant’s knowledge of the importance of physical activity in reducing breast cancer risk.

Point values assigned to all items were grounded in scientific literature, prioritizing behaviors and knowledge with the strongest evidence for breast cancer prevention. Positive health actions and accurate awareness were rewarded with higher scores, while lack of engagement, misconceptions, or inaccurate beliefs resulted in lower or negative scores.

This careful and evidence-based scoring framework enables reliable differentiation between cognitive (awareness) and behavioral dimensions, thereby enhancing the interpretation of factors affecting women’s participation in preventive mammography screening.

The MHLC-B contains 18 statements that assess generalized expectations across three dimensions: (1) Internal: The belief that an individual has control over their own health; (2) Powerful Others scale: The belief that one’s health is determined by the influence of others, especially medical personnel; and (3) Chance: the belief that health is determined by chance. Responses are rated on a 6-point Likert scale, ranging from 1 (“strongly disagree”) to 6 (“strongly agree”). Scores within each dimension range from 6 to 36 points, with higher scores indicating a stronger belief in that dimension. The MHLC-B scale is commonly used in health promotion and disease prevention programs. It is based on the premise that an internal health locus of control strengthens health-promoting behavior. Individuals with this orientation are generally more likely to engage in physical activity, reduce smoking, limit alcohol and other harmful substances, control their body weight, prevent HIV infection, and avoid other health risks (14).

The Health Behavior Inventory comprises 25 statements about health-related behaviors. Responses are rated on a 5-point Likert scale from 1 (“almost never”) to 5 (“almost always”). Based on the reported frequency of these behaviors, the intensity of four health behavior subscales is determined: (1) Healthy Habits Nutrition (HHN); (2) Preventive Behaviors (PB); (3) Positive Adjustments (PA); and (4) Health Practices (HP). The HHN subscale relates to the type of food consumed. Preventive Behaviors involve adherence to health recommendations and seeking information related to health and disease. Health Practices encompass daily routines related to sleep, recreation, and physical activity. The PA subscale includes psychological factors that support coping with stress and maintaining emotional balance. Each dimension is scored from 1 to 30 points, with higher scores indicating greater intensity of the health behavior. According to the authors, the questionnaire can support the development of preventive measures by identifying areas for improvement and tracking changes in health practices (15).

The Mini-COPE questionnaire consists of 28 statements describing typical behaviors people use in very difficult situations. It is a shortened version of the full COPE inventory, which contains 60 questions. Responses are rated on a 4-point Likert scale from 0 (“I have not been doing this at all”) to 5 (“I’ve been doing this a lot”). The tool identifies 14 distinct stress coping strategies: (1) active coping; (2) planning; (3) positive reappraisal; (4) acceptance; (5) sense of humor; (6) turning to religion; (7) seeking emotional support; (8) seeking instrumental support; (9) dealing with something else; (10) denial; (11) venting of emotions; (12) use of psychoactive substances; (13) suppression of activities; and (14) self-blame. Each scale is scored separately by summing the responses to the relevant statements and dividing the total by 2, resulting in a score range from 0 to 3 for each coping strategy. Interpretation of results is based on the diagnostic key. The inventory is intended primarily for research purposes, but can also be used in screening and preventive studies (16).

### 2.8 Study design

This study was conducted as part of the preparatory work for a doctoral dissertation that aimed to evaluate the knowledge of breast cancer risk factors among Polish women aged 45 to 69 years, to identify determinants influencing women’s participation in preventive mammography screening, and to examine the impact of demographic and psychosocial factors on delays in the diagnosis and treatment of breast cancer. The development of this project was facilitated by the approval and positive evaluation of the research proposal by the competition committee of the Polish Cancer League. Secured funding enabled the design and implementation of the research instrument. The research tool was assessed by competent judges, after which the implemented modifications were tested in a pilot study. This article reports the findings obtained from the finalized version of the survey.

The selection of the research tools in the presented study was based on the premise that women’s participation in screening is shaped by a complex interplay of cognitive, emotional, and behavioral factors, rather than solely by their level of knowledge about the disease. The use of three complementary standardized instruments: MHLC-B, HBI, Mini-COPE, alongside an author-designed sociodemographic questionnaire, enabled a multifaceted approach to the issue. This methodology enabled the assessment of both cognitive (such as knowledge and health beliefs) and emotional–behavioral components (such as coping style and health habits), which are key determinants of willingness to undergo breast cancer prophylactic examinations. This comprehensive study enabled not only the description of women’s knowledge levels and participation in preventive examinations but also the identification of the psychological mechanisms underlying health-related decisions. Such an approach significantly enhanced the analytical and cognitive value of the study’s findings. Previous research on Polish women has not addressed all of these components together, highlighting the novelty and depth of the present study’s design.

Participants were categorized into two groups: women who reported undergoing prophylactic examinations, including regular medical check-ups and/or mammography, and those who reported not participating in such screenings. This classification was based on responses to the survey question: “Do you participate in preventive screening (regular medical check-ups and/or mammography)?” According to current guidelines, regular mammography is recommended every two years for women who are eligible. For those not yet age-eligible for mammography, age-appropriate medical examinations are advised annually. The primary objective of the study was to identify factors influencing Polish women’s willingness to participate in breast cancer preventive examinations. In this context, “willingness” encompasses the various determinants examined in the study that influence women’s decisions to undergo screening. Considering the complex interplay of influences, motivations, and psychological factors, the term “willingness” was deemed the most appropriate to describe these decision-making processes.

In this study, the term “breast cancer education” encompasses any educational activity related to breast cancer. This includes formal instruction received in school addressing preventive health care, workplace training conducted in connection with Breast Cancer Awareness Month, participation in initiatives organized by cancer centers (such as White Saturday), attendance at workshops within community campaigns, lectures offered by local or regional organizations, as well as information obtained during medical consultations or through educational materials distributed as part of public awareness efforts.

In this study, the term “healthy lifestyle” is defined as a pattern of behavior aligned with national guidelines, encompassing a balanced diet, regular physical activity, sufficient sleep, avoidance of harmful substances, and adherence to other health-promoting practices.

### 2.9 Item generation, pilot testing, and scale construction

To optimize the scale construction and study design, a pilot study was conducted with a sample of 20 participants. This group was divided into two subgroups: the first subgroup (*n* = 10) received the questions organized thematically into sections addressing behavioral aspects and awareness, while the second subgroup (*n* = 10) received the same questions presented in a randomized order.

Analysis of the responses revealed that participants in the grouped question format more frequently exhibited patterned response behaviors, such as consistently selecting similar options within entire sections. This pattern may indicate the presence of automatic responses or attempts to conform to the researcher’s perceived expectations. Conversely, in the randomized question format, responses were more varied, reducing the risk of response patterning and limiting the likelihood of socially desirable responses.

Based on these observations, the final version of the survey employed a randomized question order, which was considered more cognitively neutral and better reflective of the participants’ genuine attitudes and behaviors.

### 2.10 Statistical analysis

The variables “Health Behavior” and “Prevention Awareness” were calculated from the survey questions based on the author’s key. The outcome variables of the standardized questionnaires were calculated according to the instructions and diagnostic keys provided for each tool. Statistical analysis was performed using R software (version R-4.1.2). Categorical variables were described using both absolute and relative frequencies. Numerical variables were summarized as mean ± standard deviation or median with interquartile range, depending on the normality of their distribution. Normality was assessed with the Shapiro–Wilk test and further verified by examining skewness and kurtosis. Homogeneity of variance was evaluated using Levene’s test. Comparisons between two groups were conducted using the Student’s *t*-test, Welch *t*-test, Mann–Whitney U test, Pearson’s chi-squared test, or Fisher’s exact test, as appropriate. The difference in means or medians between groups was presented with a 95% confidence interval (CI) for each numerical variable. To identify factors influencing the odds of willingness to conduct prophylactic medical examinations, a two-step logistic regression was performed. The list of factors for multivariate regression analysis was initially determined considering broader knowledge, and further mathematical selection steps were executed as follows. Selection of predictors for the multivariate model was preliminarily based on a *p*-value cut off of 0.25 (17) and subsequently on an automatic stepwise procedure. The fit of the multivariate model was assessed using Nagelkerke’s R^2^ and the Hosmer–Lemeshow test. Collinearity was checked with the variance inflation factor (VIF). Statistical significance was set at *α* = 0.05.

## 3 Results

### 3.1 Comparison of respondents who participated and who did not participate in prophylactic examinations

Statistical analysis was conducted on respondents aged 45–69 years (*n* = 407). The sample was divided into two groups: women who reported participating in prophylactic examinations, including medical checks and/or mammography (*n* = 304), and women who did not report undergoing such examinations (*n* = 103). This division served as a proxy for general willingness/unwillingness to participate in breast cancer medical examinations. In the initial phase of the analysis, these two subgroups were compared to identify factors that differentiate willingness from unwillingness to attend preventive examinations.

**Figure 1:**
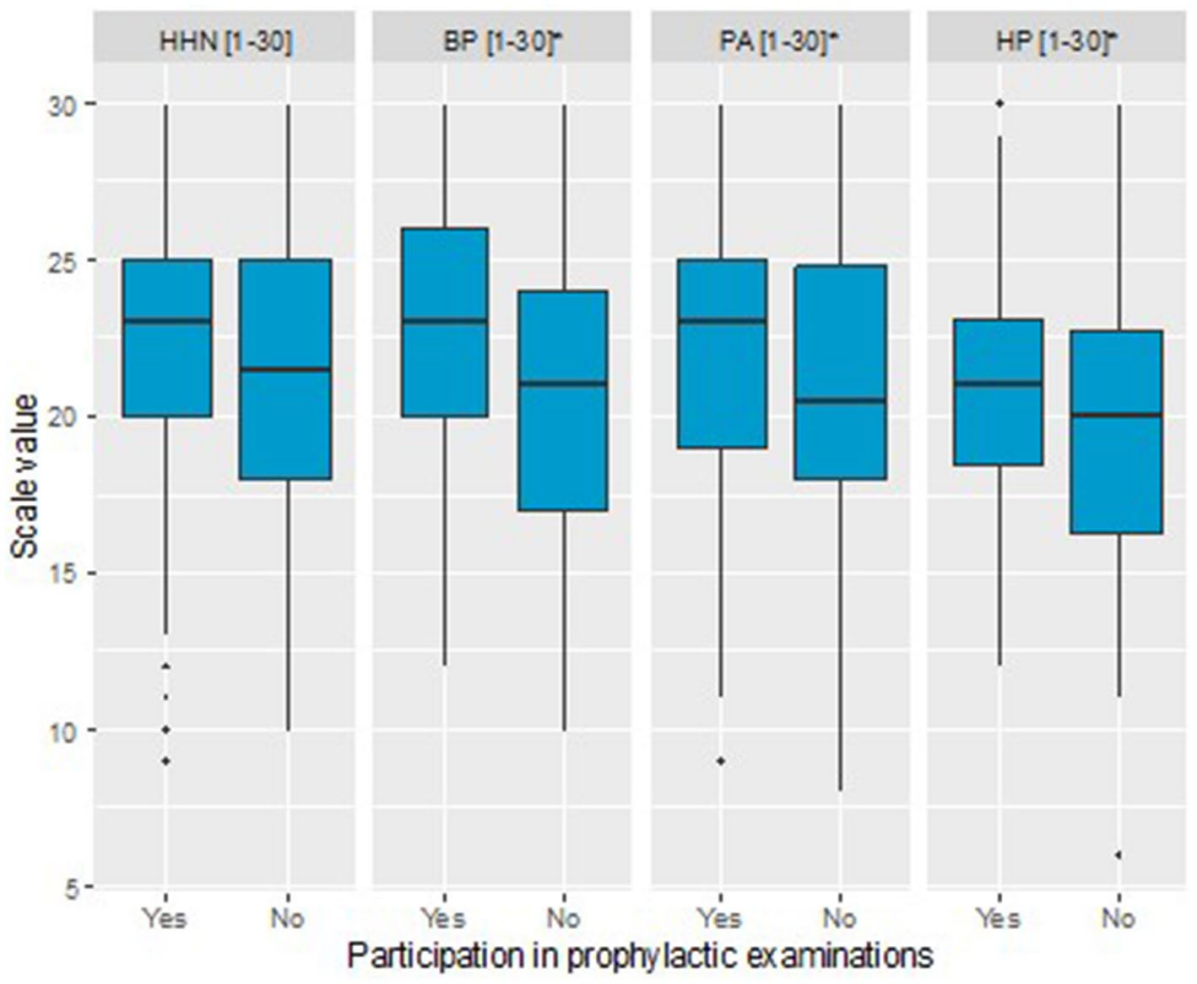
Boxplot illustrating differences in Health Behavior Inventory scales between women participating in prophylactic examinations and those not attending these examinations (*statistical difference at *p* < 0.05).

**Figure 2:**
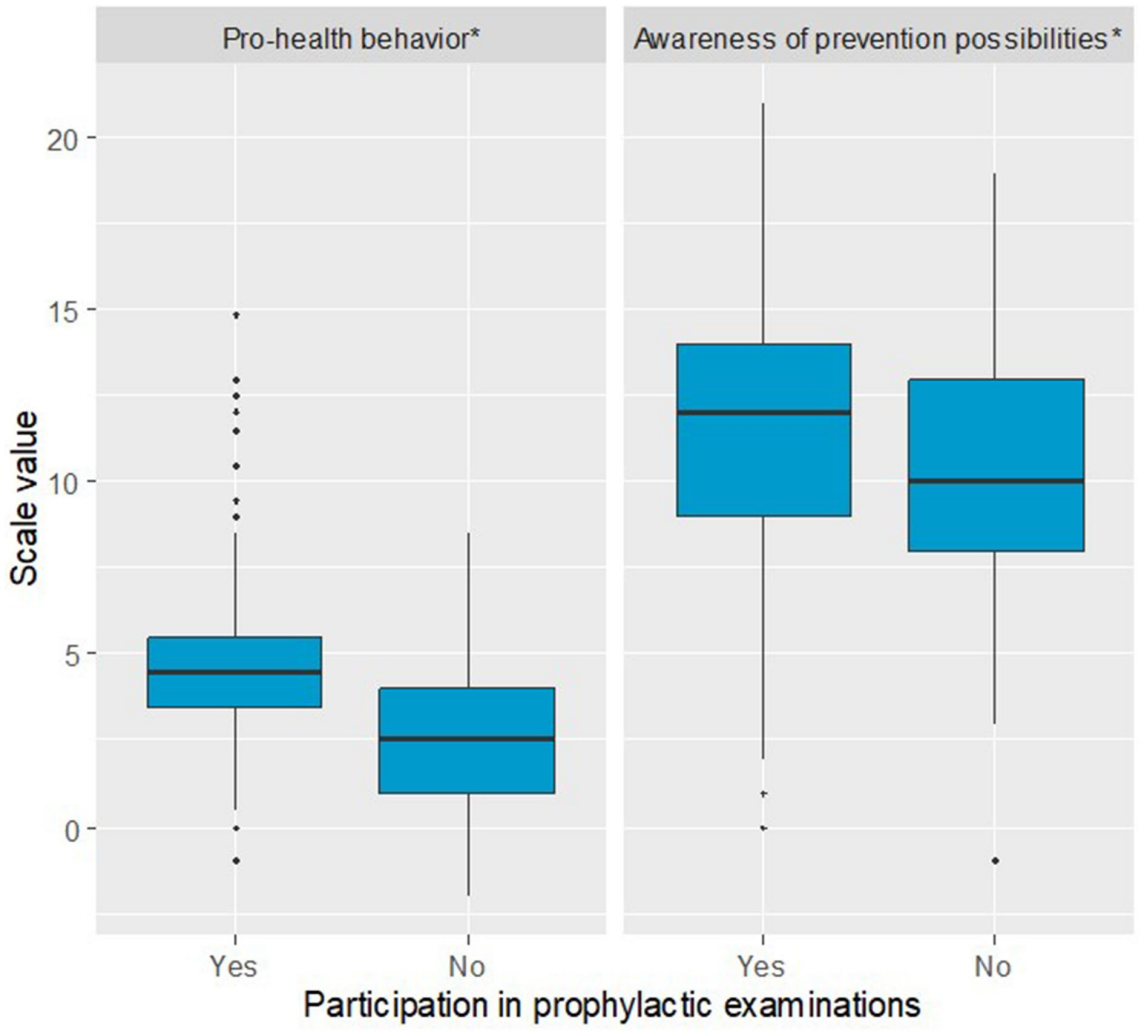
Boxplot illustrating differences in pro-health behavior and awareness of prevention possibilities scales between women participating in prophylactic examinations and those not attending these examinations (*statistical difference at *p* < 0.05).

The structure of marital status was found to significantly differentiate the groups (Pearson’s Chi-square test). Women who participated in prophylactic examinations were more often married compared to those who did not attend examinations (63.8% vs. 49.5%). Additionally, they were less frequently single/unmarried (5.6% vs. 12.6%) and less often widowed (7.6% vs. 13.6%). The proportions of women in informal relationships and those who were divorced were relatively similar between the two groups (5.6% vs. 4.9%, *n* = 5 and 17.4% vs. 19.4%, respectively). Women who participated in prophylactic examinations had significantly more children, MD = 1.00 (Mann–Whitney U test). The proportion of women who had taken hormonal contraception was significantly higher among those participating in prophylactic examinations (47.7% vs. 29.1%) (Pearson’s Chi-square test). A significant difference was also observed in the proportion of respondents who completed education on breast cancer (Pearson’s Chi-square test), with a higher percentage in the group participating in prophylactic examinations (45.4% vs. 31.1%). The mean scores on the Preventive Behaviors (PB), Positive Adjustments (PA), and Health Practices (HP) subscales of the Health Behavior Inventory questionnaire were significantly higher among respondents participating in prophylactic examinations compared to the group of non-participants (Preventive Behavior, PB: MD = 2.13, Welch *t*-test, Positive Adjustments, PA: MD = 1.03, Student’s *t*-test, Health Practices, HP: MD = 1.23, Welch *t*-test). No significant difference was found for the Healthy Habits Nutrition (HHN) subscale (Welch *t*-test). A visualization of Health Behavior Inventory scales for both groups is presented in Figure 1. Among the Mini-COPE scales, a significant difference between participants and non-participants was identified only for Seeking emotional support (Mini-COPE), MD = 0.20 (Student’s *t*-test). No significant differences were observed for the other Mini-COPE scales. Regarding the MHLC-B scales, a significant difference was found only for the Powerful Others scale, MD = 2.31, Student’s *t*-test. No significant differences were noted for the other MHLC-B, scales. Significant differences between the groups were also confirmed for both author-designed scales: Pro-Health Behavior scale (MD = 2.00, Mann–Whitney U test) and Awareness of Prevention Possibilities scale (MD = 1.26, Student’s *t*-test), with higher levels observed among women participating in prophylactic examinations in both cases, Table 1. Differences in the two scales is presented in Figure 2.

### 3.2 Factors influencing willingness for medical examinations (logistic regression analysis)

A two-step logistic regression model was developed to identify factors influencing willingness to undergo medical examination for breast cancer. The binary dependent variable was based on a yes/no question regarding participation in prophylactic examinations. Univariate regression models demonstrated that single or unmarried women had 66% lower odds of being willing to undergo examinations compared to married women, while widows had 57% lower odds compared to married women. Use of hormonal contraception was associated with twice the odds of willingness to participate. Women who did not complete education on breast cancer had 46% lower odds of willingness compared to those who had, and those who had not completed education on early detection had 41% lower odds. Three Health Behavior Inventory subscales positively influenced the odds of participation: each additional point in Preventive Behavior (PB) was associated with 12% higher odds; each point in Positive Adjustments (PA) with 6% higher odds; and each point in Health Practices (HP) with 8% higher odds. On the Mini-COPE Seeking Emotional Support scale, each additional point was associated with 35% higher odds of willingness. For the Powerful Others scale from MHLC-B, each additional point increased the odds by 7%. Higher scores on the Pro-Health Behavior scale were associated with 73% higher odds of willingness per point increase. Greater Awareness of Prevention Possibilities also increased the odds by 9% per point.

Based on the multivariate regression model, age was a significant predictor of participation in medical examinations, with each additional year associated with a 7% increase in the odds of participation. Having one additional child was associated with a 47% decrease in the odds of participation. Use of hormonal contraception nearly tripled the odds of participation. Each additional point on the Healthy Habits Nutrition (HHN) scale was associated with a 13% decrease in the odds of participation. Each additional point on the Health Practices (HP) was associated with a 9% increase in the odds of participation. Higher Pro-Health Behavior scores were strongly associated with willingness to participate, with each additional point on the Pro-Health Behavior scale doubling the odds of participation (Table 2).

**Table 2:** Logistic regression outcomes for willingness to conduct prophylactic medical examinations.

Variable	Univariate regression model			Multivariate regression model		
	OR	95% CI	*p*	OR	95% CI	*p*
Age, years	1.02	0.99–1.06	0.155	1.07	1.02–1.12	**0.005**
City size						
Village	Reference	-	-	-	-	-
City with up to 50,000 inhabitants	0.58	0.25–1.31	0.198	-	-	-
City with 50,000–150,000 inhabitants	0.76	0.30–1.90	0.557	-	-	-
City with 150,000–500,000 inhabitants	0.68	0.24–2.06	0.482	-	-	-
City with above 500,000 inhabitants	0.52	0.24–1.04	0.078	-	-	-
Education						
Vocational	Reference	-	-	-	-	-
Secondary	0.85	0.30–2.15	0.734	-	-	-
Higher	1.33	0.50–3.21	0.543	-	-	-
Marital status						
Single/unmarried	0.34	0.16–0.77	**0.008**	-	-	-
Informal relationship	0.89	0.34–2.82	0.833	-	-	-
Married	Reference	-	-	-	-	-
Divorced	0.70	0.39–1.29	0.237	-	-	-
Widow	0.43	0.21–0.92	**0.025**	-	-	-
Employment status						
Active	Reference	-	-	-	-	-
Unemployed	0.96	0.12–19.56	0.972	-	-	-
Retired	0.61	0.33–1.13	0.108	-	-	-
Pensioner	1.92	0.51–12.51	0.399	-	-	-
Number of children	1.19	0.95–1.51	0.140	0.53	0.37–0.75	**0.001**
Hormonal contraception	2.22	1.38–3.63	**0.001**	2.94	1.59–5.58	**0.001**
Healthy lifestyle	1.51	0.92–2.44	0.097	-	-	-
Knowledge on breast cancer (self-assessment)						
Very high	Reference	-	-	-	-	-
High	1.23	0.56–2.58	0.587	-	-	-
Medium	0.69	0.31–1.46	0.351	-	-	-
Low	0.94	0.23–4.80	0.938	-	-	-
Very low	0.31	0.06–1.52	0.141	-	-	-
If consulted herself if close relative has had breast cancer	1.91	0.94–3.97	0.078	-	-	-
If went through the educational process on breast cancer						
Yes	Reference	-	-	-	-	-
No	0.54	0.33–0.87	**0.013**	-	-	-
I do not know	0.52	0.16–2.02	0.303	-	-	-
If went through education on early detection of breast cancer						
Yes	Reference	-	-	-	-	-
No	0.59	0.36–0.97	**0.036**	-	-	-
I do not know	0.59	0.31–1.18	0.125	-	-	-
Medical history of breast cancer						
Yes, I am currently in the treatment process	Reference	-	-	-	-	-
Yes, currently with treatment completed	0.67	0.19–2.76	0.553	-	-	-
Yes, currently in relapse of the disease	*Inf*	-	0.983	-	-	-
No	0.61	0.29–1.20	0.172	-	-	-
Health behavior inventory						
Healthy habits nutrition, HHN [1–30]	1.05	0.99–1.10	0.097	0.87	0.79–0.94	**0.001**
Preventive behavior, PB [1–30]	1.12	1.06–1.18	**< 0.001**	1.09	1.01–1.18	**0.037**
Positive adjustments, PA [1–30]	1.06	1.00–1.12	**0.044**	-	-	-
Health practices, HP [1-30]	1.08	1.02–1.15	**0.012**	-	-	-
Mini-COPE						
Active coping [0–3]	1.21	0.86–1.70	0.270	-	-	-
Planning [0–3]	1.11	0.80–1.53	0.509	-	-	-
Positive reappraisal [0–3]	0.84	0.60–1.16	0.298	-	-	-
Acceptance [0–3]	1.00	0.72–1.38	0.997	-	-	-
Sense of humor [0–3]	0.76	0.53–1.11	0.151	-	-	-
Turning to religion [0–3]	1.10	0.89–1.37	0.396	-	-	-
Seeking emotional support [0–3]	1.35	1.02–1.79	**0.037**	1.41	0.96–2.06	0.079
Seeking instrumental support [0–3]	1.10	0.81–1.47	0.543	-	-	-
Dealing with something else [0–3]	0.93	0.69–1.26	0.653	-	-	-
Denial [0–3]	0.75	0.56–1.01	0.054	-	-	-
Venting of emotions [0–3]	0.87	0.62–1.22	0.424	-	-	-
Use of psychoactive substances [0–3]	0.80	0.5–1.14	0.206	-	-	-
Suppression of activities [0–3]	0.79	0.57–1.12	0.180	-	-	-
Self-blame [0–3]	0.83	0.61–1.13	0.236	-	-	-
MHLC-B						
Internal [6–36]	0.99	0.95–1.04	0.727	-	-	-
Powerful others [6–36]	1.07	1.03–1.12	**0.002**	-	-	-
Chance [6–36]	0.98	0.94–1.01	0.232	-	-	-
Pro-health behavior scale	1.73	1.51–2.02	**< 0.001**	2.18	1.76–2.75	**< 0.001**
Awareness of prevention possibilities scale	1.09	1.02–1.15	**0.006**	-	-	-

OR – odds ratio, CI – confidence interval. The fit of the multivariate regression model was assessed using Nagelkerke R2, which yielded an outcome of 38.3%, and the Hosmer–Lemeshow test with a *p*-value of 0.081, both confirming good fit. Collinearity was verified with VIF indicators, which were all below 1.8, confirming a low level of multicollinearity. Statistical significance at *p*<0.05 is indicated in bold font.

## 4 Discussion

### 4.1 Main findings

The authors’ analysis identified several significant factors influencing women’s participation in breast cancer screening. Notably, married women were more likely to undergo screening compared to those with other marital status. While women who participated in prophylactic examinations generally had a higher average number of children than those who did not, each additional child was paradoxically associated with a reduced likelihood of screening. Furthermore, the use of oral contraceptives was positively correlated with screening participation. Education regarding breast cancer prevention emerged as a key determinant, significantly enhancing women’s willingness to engage in preventive screening. Participants in prophylactic examinations also demonstrated markedly higher scores on three of the four subscales of the Health Behavior Inventory (HBI), indicating stronger preventive behaviors, positive health-related adjustments, and more consistent Health Practices. These women additionally expressed a greater need for emotional support and exhibited an external health locus of control, suggesting a belief that external factors influence their health outcomes. Moreover, women who underwent screening scored significantly higher on the author-designed scale. Finally, age was positively associated with screening uptake, with older women showing a greater propensity to participate in breast cancer screening. A thorough understanding of the factors influencing individuals’ willingness to participate in preventive examinations is essential for developing of effective prevention programs and public health campaigns. Such initiatives should not only raise awareness of health-promoting behaviors at both societal and individual levels but also be supported by positive experiences and facilitated through easy access to appropriate health services. In assessing behavioral patterns within a population, it is critical to consider psychological determinants, such as health locus of control, self-efficacy, and stress coping strategies, as these significantly affect decisions to engage in preventive screening. Specifically, understanding the complex interplay of psychological, behavioral, and social factors that influence women’s participation in breast cancer screening is vital to improving preventive healthcare outcomes. This discussion synthesizes current research in these fields, pinpointing existing gaps and outlining opportunities for targeted interventions to enhance screening uptake. Comparison with available research.

In the literature, fear consistently emerges as a significant reason why women do not undergo breast cancer screening. Some studies indicate that fear acts as a barrier, while others suggest that it can also motivate women to participate in medical prophylactic examinations (18–20).

Similar to our findings, research by Hanske et al. (21) and Mottram et al. (22) identified marital status as an independent predictor of breast cancer screening, while married women are significantly more likely to undergo prophylactic examinations than those who are single, divorced, or widowed.

Studies examining the impact of individual risk factors on breast cancer incidence demonstrate a correlation between the number of children born, breastfeeding, and disease risk. Anothaisintawee et al. (23) confirmed that breastfeeding reduces the risk of breast cancer by approximately 11%, and that breastfeeding for 12 months or longer increases this protective effect to 28%. Therefore, encouraging women to breastfeed, including for extended periods, is an important component of breast cancer prevention education.

Numerous studies on the prevention of another prevalent cancer, cervical, indicate that women without children are the group most likely to forgo screening. In the study by Leinonen et al. (24), women aged 45 and older without children constituted the largest group of non-participants in screening. In contrast, increasing the number of children did not affect the size of the non-participating group. Harder et al. (25) found that women with one to three children had a reduced risk of nonparticipation, while those with four or more children had an increased risk, which was attributed to potential time constraints. Notably, having one child was associated with higher screening rates, while participation decreased significantly among women with four or more children. It is important to note that these findings pertain to cytological examinations, which are generally recommended for younger women aged 25 and older.

Women who use oral contraceptives are required to visit their healthcare providers regularly to renew their prescriptions. As a result, they are more likely to visit medical facilities and may receive more frequent medical supervision, which may increase their likelihood of regular screening.

A study by Khapre et al. (26) demonstrated that health education interventions improve knowledge and the practice of breast self-examination. The study highlights that increased awareness and education lead to positive changes in attitudes and behaviors related to cancer prevention (27).

Women’s health behaviors play a crucial role in their decision to participate in preventive breast cancer screening. Women with higher health awareness and constructive coping strategies are more likely to engage in health-promoting behaviors. The results of our study are consistent with those obtained by Kłapa et al. (28), who surveyed 100 women in southern Poland using the Health Behavior Inventory and the Mini-COPE questionnaire. They found that women who maintained proper eating habits, held an optimistic outlook on life events, and actively sought health-related knowledge participated in preventive screenings more frequently and regularly in both urban and rural areas. Women employing proactive strategies, such as planning and problem-solving, were also more likely to participate in preventive screenings. In contrast, avoidance strategies, such as denial, and passivity regarding healthy eating and physical activity, were more common among women with low health awareness. Our findings confirm that health education should incorporate psychological factors, including stress coping strategies and fostering optimism, particularly in communities with lower levels of preventive knowledge.

Similarly, the study by Moghaddam-Tabrizi et al. (29) demonstrated a significant relationship between self-care and mammography, with self-care behaviors being more prevalent among those with higher perceived health motivation. This relationship has also been confirmed by Wang et al. (30), who identified healthy diet, physical exercise, and communicable diseases, including breast cancer. Azadbakht et al. (31) further supported these findings. Three key factors influence the adoption of self-care behaviors, such as mammography: personal, behavioral, and social factors (32). Personal factors refer to the values that an individual holds and prioritizes, which influence their adherence to screening behaviors for early diagnosis of the disease. Belief factors among patients are also important in disease management, as some individual perceive their health outcomes to be determined by external factors, such as luck or chance, while others believe they play a fundamental role in controlling their own health status (33). According to the results, a significant relationship was found between life priorities and participation in mammography screening.

In studies conducted on female respondents from Asian countries, including Turkey and Iran, it was confirmed that for women, a significant factor influencing participation in screening is the support of their family and spouse (34–36). Overall, the evidence highlights the importance of integrating psychological and behavioral components into health education to effectively promote preventive breast cancer screening among women.

The health locus of control facilitates the prediction of an individual’s behavioral and cognitive efforts toward their health. Studies by Brincks et al. (37) and Rodriguez et al. (38) support our findings that the individuals with an external locus of control perceive their health as determined by the actions of others; when participating in prophylactic examinations, these individuals demonstrate a high level of trust in medical care and a greater openness to seeking social support.

A study by Ghanbari et al. (39) found that willingness to attend preventive examinations increases with age among female respondents.

Analyzing health behaviors and stress-coping strategies among women who undergo preventive screenings allows for the identification of factors influencing their engagement in prevention. For this purpose, research tools such as the Health Behavior Inventory and the Mini-COPE questionnaire are commonly used, facilitating more effective planning of prevention campaigns.

### 4.2 Implications for practice

1. Increasing the availability of educational programs to promote breast cancer prevention, particularly in smaller towns and villages.
2. Implementing activities based on positive psychology to support the development of constructive strategies for coping with stress and reinforcing proper health behaviors in women from early adulthood.
3. Conducting systematic surveys of health behaviors and stress coping strategies in different social groups to better tailor prevention education and campaigns.

### 4.3 Strengths of the study

1. Due to pandemic restrictions, a significant number of respondents may have been reached by using the CAWI as a research tool. Additionally, this approach shortened the project’s duration and reduced expenses.
2. The study is unique in its multifaceted psychological approach, employing three complementary standardized questionnaires that assess both cognitive components (knowledge, health beliefs) and emotional–behavioral factors (coping style, health habits) influencing women’s willingness to undergo breast cancer preventive examinations. By addressing the significant issue of low participation rates in mammography screening and incorporating rarely studied variables such as coping style and health control beliefs, the research provides valuable insights that can inform the development of tailored health promotion strategies.
3. The findings could be used to pinpoint areas where Polish breast cancer prevention programs are lacking, indicating that preventive and educational initiatives should be tailored to the psychological characteristics of various groups of women. Such a strategy could greatly increase the efficacy of preventive programs.

### 4.4 Limitations of the study

1. The COVID-19 pandemic forced the researchers to incorporate an electronic survey, even though the initial design called for personal contact with recruited participants and the use of only the paper-and-pencil method.
2. Limited representativeness of the research group resulted from the sample’s excessively high proportion of respondents with particular characteristics (such as participants with higher education).
3. There was an excessive focus on women already participating in the screening; it would be worthwhile to study the non-participating group in more depth, taking into account systematic and psychological barriers.
4. The absence of a preregistered study protocol is acknowledged as a limitation of the present research. While this does not necessarily compromise the validity of the findings, it may introduce some risk of potential biases, such as selective reporting or unintentional flexibility in study procedures and analyses. Transparent reporting of this aspect aims to maintain research integrity and allows readers to interpret the results with appropriate caution. Future studies could benefit from registering a protocol before data collection to further enhance transparency and methodological rigor.
5. The omission of the income level and access to private healthcare services in the study posed a challenge to accurately interpreting Polish women’s attitudes toward participation in preventive screenings.
6. The questionnaire item addressing health behaviors focused solely on participants’ general subjective perceptions of their lifestyle. To avoid overburdening respondents and potentially diminishing their motivation to complete the survey, the questionnaire was intentionally kept concise and avoided excessive detail. The authors acknowledge that the broadly phrased nature of these questions may have allowed for varied interpretations among participants, representing a limitation of the present study. Future research in this area should aim to incorporate more objective measures, such as quantifying alcohol consumption in standard units over a specified period, assessing the frequency and intensity of physical activity, or measuring daily fat intake in milligrams, to enhance the precision and reliability of health behavior assessments.
7. Internal consistency of the self-designed scales was modest, reflecting their multidimensional structure and exploratory nature. For the Pro-Health Behavior scale, Cronbach’s alpha was 0.31 (standardized *α* = 0.36), while for the Awareness of Prevention Possibilities scale, Cronbach’s alpha was 0.59 (standardized α = 0.64). Further refinement and validation of these scales using larger samples are necessary in future studies.
8. Finally, the researchers recruited participants using the snowball sampling technique. Although this method makes it easier to access a previously difficult-to-reach population, it may have introduced selection bias and limited the broad applicability of the results.

## 5 Conclusion

The practical implications of the above data indicate that a woman who receives accurate information about breast cancer prevention and feels safe and comfortable with preventive examinations that are easily accessible to her is more likely to undergo regular prophylactic screenings.

## Data Availability

The original contributions presented in the study are included in the article/Supplementary material, further inquiries can be directed to the corresponding author.
