# Supplementary material for: Determinants of willingness to undergo breast cancer prophylactic examinations in Polish women
Source: Front Public Health. 2025 Sep 30;13:1583414. doi: 10.3389/fpubh.2025.1583414 (PMC12519454; doi:10.3389/fpubh.2025.1583414)
Supplement: Supplementary file 1 [file Data_Sheet_1.PDF]

Analysis of factors influencing women's participation in breast cancer screening program in Poland – the author-design section of the research tool translated for publication purposes

1. Age: ……………………

2. Size of place of residence

    a) Rural area / Village

    b) Town with up to 50,000 inhabitants

    c) Town with 50,000 to 150,000 inhabitants

    d) City with 150,000 to 500,000 inhabitants

    e) City with more than 500,000 inhabitants

3) Educational level

    a) Primary

    b) Vocational

    c) Secondary

    d) Higher

4. Marital status

    a) Single (never married)

    b) In a relationship (unmarried)

    c) Married

    d) Divorced

    e) Widowed

5. Employment status

    a) Professionally active / Employed

    b) Unemployed

    c) Retired

d) Receiving disability pension

6. During your professional activity, what was your work schedule?

    a) Daytime work

    b) Night shift work

    c) Shift work (sometimes daytime, sometimes night shifts)

    d) I have not worked

7. At what age did you experience your first menstruation? ……………………

8. How many children have you given birth to? …………………… (If 0, please proceed to Question (Q) 11)

9. How old were you when you gave birth to your first child? ……………………

10. For how long did you breastfeed (in months)? (If not at all, please enter 0; if you have multiple children, please provide the total number of months) ……………………

11. Have you ever used hormonal contraception?

a) Yes – For how long (in years)? ……………………

b) No

12. What is your current hormonal status?

a) Pre-menopausal

b) Peri-menopausal (during menopause)

c) Post-menopausal

d) I don't know

13. Have you ever used hormone replacement therapy (HRT) to alleviate menopausal symptoms?

a) Yes – For how long? ……………………

b) No

14. Do you consider yourself to lead a healthy lifestyle?

a) Yes

b) No

15. On a scale from 1 to 10, how would you rate your overall health? (1 = lowest rating, 10 = highest rating) ……………………

16. In your opinion, what factors contribute to good health? (Multiple answers possible)

a) Lifestyle

b) Physical and social environment

c) Genetic predispositions

d) Healthcare system

17. How would you assess your knowledge about breast cancer (risk factors, preventive measures, frequency of screening)?

a) Very good

b) Good

c) Average

d) Rather poor

e) Poor

18. In your opinion, which of the following factors may increase the likelihood of developing breast cancer? (Multiple answers possible)

a) Tobacco smoking

b) Alcohol consumption

c) Diet high in saturated fats (e.g., butter, cheese, lard, meat, coconut oil)

d) Prolonged breastfeeding

e) Mutations in BRCA1 and/or BRCA2 genes

f) Not having given birth

g) Long-term use of hormone replacement therapy

h) Frequent change of sexual partners

i) Wearing tight bras

j) First pregnancy after the age of 30

19. In your opinion, which of the following symptoms are indicative of breast cancer? (Multiple answers possible)

a) Dizziness

b) Shortness of breath

c) Changes in breast skin texture (wrinkling, retraction, ulceration)

d) Nausea and/or vomiting

e) Changes in the nipple (ulceration, retraction, discharge)

f) Enlargement of lymph nodes

g) Breast pain

h) Lumps/thickenings that appear before menstruation and decrease or disappear afterward

20. Please select the statements that you believe to be true. (Multiple answers possible)

a) Breast cancer is incurable.

b) The earlier breast cancer is detected, the higher the chances of complete recovery.

c) If breast cancer occurred in a close relative (mother, aunt, or sister), I will also develop the disease.

d) A proper lifestyle (diet, physical activity) can prevent the development of breast cancer.

21. Have there been any cases of breast cancer among women in your immediate family (e.g., grandmother, mother, sister, daughter)?

a) Yes

b) No (Please proceed to Q 24)

22. Knowing that a close relative has had breast cancer, did you consult a doctor to assess your own risk of developing the disease?

a) Yes

b) No

23. In your opinion, did the doctor clearly explain what should be done to prevent the disease?

a) Yes

b) No

24. Have you ever received education on breast cancer prevention?

a) Yes

b) No

c) I don't know

25. In your opinion, where should women receive comprehensive information about breast cancer (risk factors, prevention, early detection) to achieve the best prevention outcomes? (Multiple answers possible)

a) Primary school

b) Secondary school

c) University

d) Workplace

e) Family (e.g., mother of an adolescent daughter, older sister)

f) General practitioner

g) Gynecologist

h) Media, public campaigns, brochures, lectures

26. Have you ever received education on early detection of breast cancer?

a) Yes – How old were you at that time? ....

b) No

c) I don't know

27. Do you believe that the education you received sufficiently increased your knowledge about prevention and/or early detection of breast cancer?

a) Yes

b) No

c) I don't know

28. What form did the educational activities you participated in take? (Multiple answers possible)

a) Conversation with a primary care physician

b) Conversation with a gynecologist

c) Conversation with a nurse / midwife

d) Obtaining information from informational articles in the press / internet

e) Obtaining information from informational brochures

f) Media information campaigns

g) Educational sessions, e.g., lectures, seminars

h) Not applicable

29. Do you participate in preventive screening examinations (regular medical check-ups and/or mammography)?

a) Yes – How many months ago did you have a mammogram? .... (Please proceed to Q 31)

b) No (Please proceed to Q 30)

30. If not, what are the reasons? (Multiple answers possible)

a) I consider myself healthy.

b) Due to fear / anxiety about illness.

c) Due to fear / anxiety related to visiting a doctor.

d) Due to embarrassment and shame associated with the examination.

e) I consider myself too young for such examinations.

f) Due to lack of time.

g) I do not know where to have such an examination done.

h) I do not consider this examination important.

i) To undergo this examination, I would have to undertake a long trip, which I cannot afford.

j) I do not have regular access to healthcare.

31. Do you know how to perform a proper breast self-examination?

a) Yes

b) No

32. Who or what motivated you to perform breast self-examination? (Multiple answers possible)

a) Family (e.g., mother, sister)

b) Friend / acquaintance

c) Partner

d) Primary care physician

e) Gynecologist

f) Nurse

g) Midwife

h) Illness of a close person

i) Influence of social education (campaigns)

j) Influence of school education

33. How often do you perform breast self-examination?

a) Once a month

b) Several times a year

c) Once every six months

d) Never, because I do not know how to do it properly

e) Never, because I do not consider it important

f) Never, for health reasons (e.g., I have undergone bilateral mastectomy)

34. In your opinion, how often should women aged 40-49 undergo regular medical examinations for early detection of potential breast cancer lesions?

a) Once a year

b) Once every 2 years

c) Once every 3 years

d) Once every 5 years

35. In your opinion, how often should women aged 50 to 69 undergo mammography screening?

a) Once a year

b) Once every 2 years

c) Once every 3 years

d) Once every 5 years

36. What do you think may constitute barriers to preventive actions that could reduce the risk of developing breast cancer? (Multiple answers possible)

a) Lack of financial resources (high cost of examination)

b) Long waiting time for the examination

c) Large distance between place of residence and appropriate medical facility

d) Lack of time

e) Lack of trust in doctors

f) Reluctance to undergo screening examinations

g) Lack of adequate knowledge

37. Please select ALL answers that apply to your situation:

a) I regularly consume alcohol

b) I usually consume foods high in fats

c) I am currently undergoing treatment for cancer other than breast cancer

d) I have had cancer other than breast cancer in the past (treatment completed)

e) I am moderately physically active (e.g., walking, recreational cycling/swimming)

f) I regularly engage in intensive physical activity (e.g., running, competitive sports such as basketball, aerobics)

g) I am not physically active

h) I undergo breast ultrasound every 1-2 years

38. Have you ever been diagnosed with breast cancer?

a) Yes, I am currently undergoing treatment (Please proceed to Question 39)

b) Yes, in the past, treatment has been completed (Please proceed to Question 39

c) Yes, in the past, I was treated but the disease recurred (Please proceed to Question 39)

d) No (Please proceed to the questions in Section 2[1] on the next page)

39. How was your breast cancer detected?

a) Incidentally during the ultrasound examination

b) Incidentally during mammography

c) During a medical examination (other than mammography or ultrasound) as part of a scheduled visit

d) During a medical examination following the appearance of concerning symptoms (e.g., breast pain, nipple discharge)

e) Through breast self-examination

f) By a partner

40. At what stage was your breast cancer diagnosed?

a) 0

b) I

c) II

d) III

e) IV

f) I don't know

41. How old were you at the time of your breast cancer diagnosis? ....

---

[1] Section 2 consists of standardized questionnaire that are copyrighted and their release is against the Publisher's policy.
