# Supplementary material for: Determinants of willingness to undergo breast cancer prophylactic examinations in Polish women
Source: Front Public Health. 2025 Sep 30;13:1583414. doi: 10.3389/fpubh.2025.1583414 (PMC12519454; doi:10.3389/fpubh.2025.1583414)
Supplement: Supplementary file 2 [file Data_Sheet_2.PDF]

Table 1. The calculation key corresponding to the author-designed questionnaire.

Pro-health behavior scale	Awareness of prevention possibilities scale
**Number of children** (+1 point per child)	**Awareness of breast cancer risk factors** – smoking, alcohol, high in fat diet, positive outcome of BRCA1 or BRCA2, not giving birth, long-term hormonal replacement therapy, 1st pregnancy after 30 years (+0.5 point for each factor)
**Length of breastfeeding** (+1 point for each 12 months)	**Awareness of breast cancer symptoms** – change in breast skin structure, changes of nipple, swollen lymph nodes (+0.5 point for each symptom)
**Participation in prophylactic examination** (+1 point if within last 2 years, +0.5 point if earlier than 2 years ago, -1 point for lack of participation)	**Belief that breast cancer is incurable** (-1 point)
**Breast self-assessment** (+1 point if monthly, +0.5 point if up to twice a year, -1 point for importance not admitted)	**Awareness that the sooner the diagnosis of breast cancer, the better prognosis** (+1 point)
**Regular consumption of alcohol** (-1 point)	**Awareness that healthy lifestyle is a breast cancer prevention behavior** (+1 point)
**High in fat diet** (-1 point)	**If consulted herself if close relative has had breast cancer** (+1 point if yes, -1 point if no)
**Moderate physical activity** (+1 point)	**If went through educational on breast cancer prevention** (+1 point if yes)
**Regular intensive physical activity** (+1 point)	**If went through educational on early breast cancer detection** (+1 point if yes)
**No physical activity** (-1 point)	**Participation in prophylactic examination** (+1 point if within last 2 years, +0.5 point if earlier than 2 years ago, -1 point for lack of participation)
**Breast ultrasound every 1-2 years** (+1 point)	**If declares ability to breast self-assessment** (+1 point if yes, -1 point if no)
	**Breast self-assessment** (+1 point if monthly, +0.5 point if up to twice a year, -1 point for importance not admitted)
	**Frequency of breast prophylactic examination at age 40-49 years** (+1 point if annually)
	**Frequency of mammogram examination at age 50-69 years** (+1 point if once in two years)
	**Regular consumption of alcohol** (-1 point)
	**Consumption of high in fat meals** (-1 point)
	**Moderate physical activity** (+1 point)
	**Regular intensive physical activity** (+2 point)

Pro-health behavior scale	Awareness of prevention possibilities scale
	**No physical activity** (-1 point)
	**Breast ultrasound every 1-2 years** (+1 point)
