# Supplementary material for: Determinants of willingness to undergo breast cancer prophylactic examinations in Polish women
Source: Front Public Health. 2025 Sep 30;13:1583414. doi: 10.3389/fpubh.2025.1583414 (PMC12519454; doi:10.3389/fpubh.2025.1583414)
Supplement: Supplementary file 3 [file Data_Sheet_3.pdf]

Table 1. The STROBE checklist.

	Item No	Recommendation	Text position (page (p.) and lines numbers (ln.))
**Title and abstract**	1	(*a*) Indicate the study's design with a commonly used term in the title or the abstract	p. 3, ln. 227-274
		(*b*) Provide in the abstract an informative and balanced summary of what was done and what was found	p. 1, ln. 77-96
**Introduction**			
Background/rationale	2	Explain the scientific background and rationale for the investigation being reported	p. 1-3, ln. 105-268
Objectives	3	State specific objectives, including any prespecified hypotheses	p. 3, ln. 272-274
**Methods**			
Study design	4	Present key elements of study design early in the paper	p. 5, ln. 715-781
Setting	5	Describe the setting, locations, and relevant dates, including periods of recruitment, exposure, follow-up, and data collection	p. 3, ln. 279-293, 321-328
Participants	6	(*a*) Give the eligibility criteria, and the sources and methods of selection of participants	p. 3, ln. 279-291
Variables	7	Clearly define all outcomes, exposures, predictors, potential confounders, and effect modifiers. Give diagnostic criteria, if applicable	p. 6-8, ln. 606-617, 672-679, 682-696, 701-712, 717-729, 807-832
Data sources/ measurement	8*	For each variable of interest, give sources of data and details of methods of assessment (measurement). Describe comparability of assessment methods if there is more than one group	p. 6, ln. 584-605
Bias	9	Describe any efforts to address potential sources of bias	p. 3-5, ln. 331-508
Study size	10	Explain how the study size was arrived at	p. 3, ln. 281-282, 298-318
Quantitative variables	11	Explain how quantitative variables were handled in the analyses. If applicable, describe which groupings were chosen and why	p. 8, ln. 812-815
Statistical methods	12	(*a*) Describe all statistical methods, including those used to control for confounding	p. 8, ln. 812-831

		(*b*) Describe any methods used to examine subgroups and interactions	p. 8, ln. 812-831
		(*c*) Explain how missing data were addressed	N/A
		(*d*) If applicable, describe analytical methods taking account of sampling strategy	N/A
		(*e*) Describe any sensitivity analyses	N/A
**Results**			
Participants	13*	(a) Report numbers of individuals at each stage of study—eg numbers potentially eligible, examined for eligibility, confirmed eligible, included in the study, completing follow-up, and analysed	p. 3, 8, ln. 281-282, 841-845
		(b) Give reasons for non-participation at each stage	N/A
		(c) Consider use of a flow diagram	N/A
Descriptive data	14*	(a) Give characteristics of study participants (eg demographic, clinical, social) and information on exposures and potential confounders	p. 8, ln. 851-891; Table 1
		(b) Indicate number of participants with missing data for each variable of interest	N/A
Outcome data	15*	Report numbers of outcome events or summary measures	p. 8, ln. 841-845; Table 1
Main results	16	(*a*) Give unadjusted estimates and, if applicable, confounder-adjusted estimates and their precision (eg, 95% confidence interval). Make clear which confounders were adjusted for and why they were included	Table 2
		(*b*) Report category boundaries when continuous variables were categorized	N/A
		(*c*) If relevant, consider translating estimates of relative risk into absolute risk for a meaningful time period	N/A
Other analyses	17	Report other analyses done—eg analyses of subgroups and interactions, and sensitivity analyses	N/A
**Discussion**			
Key results	18	Summarise key results with reference to study objectives	p. 10, ln. 1054-1094
Limitations	19	Discuss limitations of the study, taking into account sources of potential bias or imprecision. Discuss both direction and magnitude of any potential bias	p. 13, ln. 1437-1491
Interpretation	20	Give a cautious overall interpretation of results considering objectives, limitations,	p. 10-13, ln. 1095-1491

		multiplicity of analyses, results from similar studies, and other relevant evidence	
Generalisability	21	Discuss the generalisability (external validity) of the study results	p. 13, ln. 1398-1434, 1494-1500
**Other information**			
Funding	22	Give the source of funding and the role of the funders for the present study and, if applicable, for the original study on which the present article is based	p. 14, ln. 1531-1536

*Give information separately for exposed and unexposed groups.
"N/A" – "not applicable".

Reference: STROBE. Strengthening the reporting of observational studies in epidemiology. https://www.strobe-statement.org/. [Accessed April 24, 2025].
